# Supplementary material for: Assessing document section heterogeneity across multiple electronic health record systems for computational phenotyping: A case study of heart-failure phenotyping algorithm
Source: PLoS One. 2023 Mar 31;18(3):e0283800. doi: 10.1371/journal.pone.0283800 (PMC10065225; doi:10.1371/journal.pone.0283800)
Supplement: S1 Table — aOne clinical document standard of IC Chart Electronic Health Record (EHR) consists of sections ‘S,’ ‘O’, ‘A,’ and ‘P.’; The “Impression and Plan” section of the Cerner EHR, corresponds to “the Assessment and Plan” section of the General Electronic Centricity (GEC) EHR, which contains information on the “Problem,” “Medications,” and other sections; “Chief Complaint and Reason for Visit” sections of the Epic EHR are similar to “Problem” sections of the GEC EHR. (DOCX) [file pone.0283800.s001.docx]

**S1 Table.** **Differentiation of corresponding sections and subsections among corpora^a^**

| IC Chart | **P**: 1. We will refer to Cardiology for further evaluation of her CHF  with diastolic dysfunction (in particular to assess whether  or not patient should continue on digoxin).  2. Recommended followup chest x-ray, but patient and son decline  at this time.  3. Once again, advised that patient be seen in Neurology Clinic  and Allergy Clinic (for evaluation of her reported  forgetfulness and for evaluation of possible allergic  reaction with tongue and lip swelling); but, again, patient  and son decline at this time.  4. Code status DNR/DNI per patient request.  5. Continue using all medications as written for now.  6. Will follow up in clinic in six months at which time full  health maintenance exam including vaginal/pelvic exams with  Pap will be completed. We will also schedule a mammogram at  that time. |
| --- | --- |
| Cerner | **Impression and Plan**  Diagnosis  Hemarthrosis of shoulder (ICD10-CM M25.019, Discharge, Medical)  Plan  Prescriptions: Launch prescriptions  Pharmacy:  acetaminophen-HYDROcodone 325 mg-5 mg oral tablet (Prescribe): 1-2 tab(s), Oral, q4hr (interval), for 5 day(s), PRN: as needed for pain, 20 tab(s), 0 Refill(s)  .  Patient was given the following educational materials: Shoulder Pain.  Follow up with: ***_NAME_*** Within 1 week Call for follow up appointment  Return if symptoms worsen.  Orders: Launch Orders  Patient Care:  Sling (Order): ***_DATE_*** 8:46 CDT, Place Sling On Affected Extremity |
| Epic | **Chief Complaint**  Patient presents with  Hyperlipidemia  Hyperglycemia  Hypothyroidism  Congestive Heart Failure  Aortic valve stenosis  ….  **Assessment/Plan** Diagnoses and all orders for this visit:  Hypercholesterolemia  - Comprehensive metabolic panel; Future  - Lipid panel; Future  - Thyroid Function Cascade; Future  - atorvastatin (LIPITOR) 40 MG tablet; Take 1 tablet (40 mg total) by mouth  1 (one) time each day in the evening  Hyperglycemia  - Comprehensive metabolic panel; Future  - Urinalysis with reflex microscopic; Future  - Hemoglobin A1c; Future  Hypothyroidism, unspecified type  - Lipid panel; Future  - Thyroid Function Cascade; Future  - levothyroxine (SYNTHROID) 100 MCG tablet; Take 1 tablet (100 mcg total) by  mouth 1 (one) time each day in the morning Alone with water on an empty stomach  Chronic systolic congestive heart failure  - CBC auto differential; Future  - Comprehensive metabolic panel; Future  - Thyroid Function Cascade; Future  - triamterene-hydroCHLOROthiazide (MAXZIDE-25) 37.5-25 MG per tablet; Take 2  tablets by mouth 1 (one) time each day Skip a dose if light headed  Nonrheumatic aortic valve stenosis  - CBC auto differential; Future |

^a^One clinical document standard of IC Chart Electronic Health Record (EHR) consists of sections ‘S,’ ‘O’, ‘A,’ and ‘P.’; The “Impression and Plan” section of the Cerner EHR, corresponds to “the Assessment and Plan” section of the General Electronic Centricity (GEC) EHR, which contains information on the “Problem,” “Medications,” and other sections; “Chief Complaint and Reason for Visit” sections of the Epic EHR are similar to “Problem” sections of the GEC EHR.
