# Supplementary material for: Assessing document section heterogeneity across multiple electronic health record systems for computational phenotyping: A case study of heart-failure phenotyping algorithm
Source: PLoS One. 2023 Mar 31;18(3):e0283800. doi: 10.1371/journal.pone.0283800 (PMC10065225; doi:10.1371/journal.pone.0283800)
Supplement: Attachment — Submitted filename: ResponseToReviewers.docx [file pone.0283800.s002.docx]

Journal Requirements:

When submitting your revision, we need you to address these additional requirements.

1. Please ensure that your manuscript meets PLOS ONE's style requirements, including those for file naming. The PLOS ONE style templates can be found at 
<https://journals.plos.org/plosone/s/file?id=wjVg/PLOSOne_formatting_sample_main_body.pdf> and 
<https://journals.plos.org/plosone/s/file?id=ba62/PLOSOne_formatting_sample_title_authors_affiliations.pdf>

Response: As you suggested, we have revised the manuscript to meet PLOS ONE’s style requirement.

2. Please provide additional details regarding participant consent. In the ethics statement in the Methods and online submission information, please ensure that you have specified (1) whether consent was informed and (2) what type you obtained (for instance, written or verbal, and if verbal, how it was documented and witnessed). If your study included minors, state whether you obtained consent from parents or guardians. If the need for consent was waived by the ethics committee, please include this information.

If you are reporting a retrospective study of medical records or archived samples, please ensure that you have discussed whether all data were fully anonymized before you accessed them and/or whether the IRB or ethics committee waived the requirement for informed consent. If patients provided informed written consent to have data from their medical records used in research, please include this information.

Response: We added sentences, “Data sharing is restricted by the Mayo Clinic and Olmsted Medical Center Institutional Review Boards for human subject research because data from three different electronic health records contain sensitive patient information. Machine learning models will be available on request to rstnlp@mayo.edu that will provide data access request document.” in line 358.

3. Please note that PLOS ONE has specific guidelines on code sharing for submissions in which author-generated code underpins the findings in the manuscript. In these cases, all author-generated code must be made available without restrictions upon publication of the work. Please review our guidelines at <https://journals.plos.org/plosone/s/materials-and-software-sharing#loc-sharing-code> and ensure that your code is shared in a way that follows best practice and facilitates reproducibility and reuse.

Response: We added sentences, “Data sharing is restricted by the Mayo Clinic and Olmsted Medical Center Institutional Review Boards for human subject research because data from three different electronic health records contain sensitive patient information. Machine learning models will be available on request to rstnlp@mayo.edu that will provide data access request document.” in line 358.

4. Thank you for stating the following financial disclosure: 
 "This work was supported by National Institutes of Health grant numbers TR002062 (HL), LM011934 (HL), and HL136659 (SJB). This study was made possible using the resources of the Rochester Epidemiology Project, which was supported by the National Institute on Aging of the National Institutes of Health under Award Numbers AG034676 (Walter Rocca) and AG052425(Walter Rocca)."

Please state what role the funders took in the study.  If the funders had no role, please state: "The funders had no role in study design, data collection and analysis, decision to publish, or preparation of the manuscript." 
If this statement is not correct you must amend it as needed. 
Please include this amended Role of Funder statement in your cover letter; we will change the online submission form on your behalf.

Response: We have a “Contributorship statement” for the role of funders (HL and SJB) in line 374. We added a phrase, “We would like to thank Donna Ihrke and Ellen E. Koepsell for conducting annotations**, as well as Dr. Walter Rocca for providing the dataset**.” for the role of Dr. Walter Rocca in line 354.

5. In your Data Availability statement, you have not specified where the minimal data set underlying the results described in your manuscript can be found. PLOS defines a study's minimal data set as the underlying data used to reach the conclusions drawn in the manuscript and any additional data required to replicate the reported study findings in their entirety. All PLOS journals require that the minimal data set be made fully available. For more information about our data policy, please see <http://journals.plos.org/plosone/s/data-availability>.

"Upon re-submitting your revised manuscript, please upload your study’s minimal underlying data set as either Supporting Information files or to a stable, public repository and include the relevant URLs, DOIs, or accession numbers within your revised cover letter. For a list of acceptable repositories, please see <http://journals.plos.org/plosone/s/data-availability#loc-recommended-repositories>. Any potentially identifying patient information must be fully anonymized.

Important: If there are ethical or legal restrictions to sharing your data publicly, please explain these restrictions in detail. Please see our guidelines for more information on what we consider unacceptable restrictions to publicly sharing data: <http://journals.plos.org/plosone/s/data-availability#loc-unacceptable-data-access-restrictions>. Note that it is not acceptable for the authors to be the sole named individuals responsible for ensuring data access.

We will update your Data Availability statement to reflect the information you provide in your cover letter.

Response: We added sentences, “Data sharing is restricted by the Mayo Clinic and Olmsted Medical Center Institutional Review Boards for human subject research because data from three different electronic health records contain sensitive patient information. Machine learning models will be available on request to rstnlp@mayo.edu that will provide data access request document.” in line 358.

6. Your ethics statement should only appear in the Methods section of your manuscript. If your ethics statement is written in any section besides the Methods, please move it to the Methods section and delete it from any other section. Please ensure that your ethics statement is included in your manuscript, as the ethics statement entered into the online submission form will not be published alongside your manuscript.

Response: We moved a sentence, “The study has appropriate approvals by the Mayo Clinic and Olmsted Medical Center Institutional Review Boards (IRBs Mayo 13-009317, Mayo 17-008818, and OMC 053-OMC-17).” in the Methods section in line 142. And we deleted the “ethics statement”.

Additional Editor Comments:
Both reviewers noted the value of the study described in this manuscript. However, they both also mentioned some areas for clarification an improvement. In particular, additional description of the methodology and experimental design as well as some additional discussion of previous work in this area could be beneficial.

Response: Thank you. We have revised the manuscript, and hopefully, the revision clarifies all questions for improvement.

[Note: HTML markup is below. Please do not edit.]

Reviewers' comments:

Reviewer's Responses to Questions

**Comments to the Author**

1. Is the manuscript technically sound, and do the data support the conclusions?

The manuscript must describe a technically sound piece of scientific research with data that supports the conclusions. Experiments must have been conducted rigorously, with appropriate controls, replication, and sample sizes. The conclusions must be drawn appropriately based on the data presented.

Reviewer #1: Partly

Reviewer #2: Partly

Response: We have revised the manuscript, and hopefully, the revision makes the research technically sound.

2. Has the statistical analysis been performed appropriately and rigorously?

Reviewer #1: No

Reviewer #2: No

Response: We followed the best practices in classification tasks and reported confidence intervals (Table 3). We also responded to the issues raised, including removing the transferability measure.

3. Have the authors made all data underlying the findings in their manuscript fully available?

The [PLOS Data policy](https://nam12.safelinks.protection.outlook.com/?url=http%3A%2F%2Fwww.plosone.org%2Fstatic%2Fpolicies.action%23sharing&data=05%7C01%7CMoon.Sungrim%40mayo.edu%7C44fe22cb3ac1437853de08dac7270263%7Ca25fff9c3f634fb29a8ad9bdd0321f9a%7C0%7C0%7C638041267598666228%7CUnknown%7CTWFpbGZsb3d8eyJWIjoiMC4wLjAwMDAiLCJQIjoiV2luMzIiLCJBTiI6Ik1haWwiLCJXVCI6Mn0%3D%7C3000%7C%7C%7C&sdata=yiKfIPcnoe6ESPAXE9DndP5hNT9zBByvW4e4SqCXXco%3D&reserved=0) requires authors to make all data underlying the findings described in their manuscript fully available without restriction, with rare exception (please refer to the Data Availability Statement in the manuscript PDF file). The data should be provided as part of the manuscript or its supporting information, or deposited to a public repository. For example, in addition to summary statistics, the data points behind means, medians and variance measures should be available. If there are restrictions on publicly sharing data—e.g. participant privacy or use of data from a third party—those must be specified.

Reviewer #1: No

Reviewer #2: No

Response: We added sentences, “Data sharing is restricted by the Mayo Clinic and Olmsted Medical Center Institutional Review Boards for human subject research because data from three different electronic health records contain sensitive patient information. Machine learning models will be available on request to rstnlp@mayo.edu that will provide data access request document.” in line 358.

4. Is the manuscript presented in an intelligible fashion and written in standard English?

PLOS ONE does not copyedit accepted manuscripts, so the language in submitted articles must be clear, correct, and unambiguous. Any typographical or grammatical errors should be corrected at revision, so please note any specific errors here.

Reviewer #1: Yes

Reviewer #2: Yes

5. Review Comments to the Author

Please use the space provided to explain your answers to the questions above. You may also include additional comments for the author, including concerns about dual publication, research ethics, or publication ethics. (Please upload your review as an attachment if it exceeds 20,000 characters)

Reviewer #1: 1. You may need to cite or compare this work as it utilized advanced machine learning models to leverage heterogeneous EHR "HCET: Hierarchical Clinical Embedding With Topic Modeling on Electronic Health Records for Predicting Future Depression"

Response 1.1: We agreed on the significance of this paper, so we added it in the background, such as “**Beyond overcoming the heterogeneity of diverse data types in EHR using a hierarchical embedding-based model, these approaches offer** the potential to detect sections.” in line 105.

2. How did choose the threshold for the F-measure? It is more robust to show average precision or precision-recall area under the curve instead of F-measure as the latter depends on the threshold you choose

Response 1.2: Thank you for raising this comment. We used the default parameter provided by the python package and the traditional NLP performance metric, F-measure, to measure the context classification performance. The primary message we hoped to deliver is not about obtaining the best performance but rather assessing the heterogeneity of different EHRs. Our main purpose of the study is to check if a classifier trained with data from one EHR can be used for context classification of unstructured data from other EHRs. We discussed this as a limitation of the study. One sentence is “**For random forest model, we used the default threshold as 0.5.**” in line 192. And the other sentence indicates our experience limitation, such as “We did not thoroughly compare various ML algorithms and other BERT-based approaches for section classification tasks **or exhausting parameter optimizations for the RF and BERT models**.” in line 333.

3. What's the ratio between case and non-case in for the training set of GEC? Because the ratio of cases is above 90%in all test sets, which is quite high and not reflect the true ratio in reality for the classifier. You need to resample the test set

Response 1.3: As you suggested, we added the sample size of the train set in GEC in Table 2 in line 245. The high ratio of cases in train and test sets is because we applied the HF-phenotype algorithm on clinical notes (Fig 1). The purpose of the NLP-based HF-phenotype algorithm is to find a clinical section containing phenotype (heart-failure incidents) relevant sentences which belong to six particular sections.

4. Since both algorithms generated high F1 score (above 0.8) in the final binary outcome of HF phenotyping-relevant, you can draw the conclusion of transferability instead of focusing on each subsection.

Response 1.4: Thank you for your suggestion. We added one sentence, “**In this study, we achieved an above 0.8 F1-score for detecting HF phenotyping-relevant sections that present high transferability**.” in line 294.

5. In line 79, please remove "embedding-based" as random forest can take any input types. Writing in this way would make readers misunderstand that it inherently takes embeddings as the input.

Response 1.5: We removed “with sentence embedding-features” in line 82 (initially line 79), as suggested.

6. In line 177, it should be true positive instead of false positive.

Response 1.6: We revised the sentence to remove misleading context, such as “**Meanwhile**, if the “History of Present Illness” classifier labels any of the three sentences as true, then the text segment is labeled as the “History of Present Illness” section, which is a case of false positive for the “History of Present Illness” classifier **because the original sentences in the silver standard belong to the problem section.**” in line 181 (initially line 177).

Reviewer #2: In this paper the authors evaluated the use of machine learning algorithms to detect heterogeneity for heart failure phenotyping across different EHR systems. Specifically, the authors used RF and BERT to classify sections given a segment of multiple sentences from an EHR note. The authors defined a transferability metric and showed that the models trained on one EHR system performed differently on other EHR-systems and the transferability score also varied across different EHR systems.

Strengths:
=======
1. The paper is well-written and well-structured.

2. The authors demonstrated the proper use of multiple EHR databases.

3. The authors showed the complexity and heterogeneity of heart-failure relevant section names across different EHR systems.

Response 2.0: Thank you for those positive comments.

Weaknesses:
=======
1. The algorithm for the section classification is not clear. Why the authors preferred multiple separate classifiers instead of a single multi-class or multi-label (if a text segment belongs to multiple sections) classifier? Why all sentences in a text segment were classified separately (this weakens contextualized language models such as BERT)? How was the model training performed for these separate classifiers?

Response 2.1: This is a valuable comment for us. We added a reason, such as “**Note that we choose binary classifiers due to the low performance of a multi-class or multi-label classifier.**” In line 196. We also added one sentence, “**To fair comparison, we used the same chunk of the sentence set for training both BERT and RF models; however, this setting may lead to the disadvantage of BERT, which is a contextualized language model.”** in line 336.

2. The transferability metric is not well-defined. A model with similar F-scores on two different test sets does not necessarily mean the system is transferable. A better evaluation method is to train a model (say, BERT) on a training set of the “to-be-tested” EHR system (IC Chart, Cerner, or Epic), test it on its hold-out test set and then compare this performance with the performance of a similar model trained on the source EHR system (GEC EHR). This gives a good indication of transferability, also known as domain adaptation.

Response 2.2: We agreed with the reviewer’s definition of transferability. So, we removed the “transferability score” in the method (line 209) and the result, including Table 4 (line 266), to remove misled definition of transferability. Our primary study objective is to quantify the heterogeneity across EHR systems and point out challenges faced by context-based computational phenotyping algorithms.

3. The authors should explain why difference in section names among different EHR systems is analogous to overall heterogeneity. The paper title suggests overall heterogeneity and do not indicate that the study is limited to heart failure phenotyping algorithm. Either these should be explained in the paper body or the title should be revised.

Response 2.3: As suggested, we added one sentence and revised two phrases, “**However, distinct EHR systems often use heterogeneous documentation (e.g., the lexical variants to describe the same clinical section, one major section that matches multiple granular document subsections in other EHR systems), leading to the challenge of identifying corresponding sections between separate EHRs for phenotype algorithms.** In this study, we explored the use of embedding-based machine learning (ML) classifiers **to detect corresponding sections among different EHRs,** which were trained using a labeled corpus automatically extracted from the GEC system and evaluated using a blind test set sampled across four EHR systems **using HF phenotyping algorithm as a case study.**” In line 75. Also, we revised the title “Assessing document section heterogeneity across multiple electronic health record systems for computational phenotyping**: A case study of heart-failure phenotyping algorithm.**”

4. The term ‘case’ was not defined for table2.

Response 2.4: As you suggested, we defined the term ‘case’ for Table 2, such as “**^a^a sentence chunk containing the description of a heart failure incident**” for ‘case’ and “**^b^a sentence chunk not about a heart failure incident**” for ‘non-case’ in Table 2 at lines 247-248.

6. PLOS authors have the option to publish the peer review history of their article ([what does this mean?](https://nam12.safelinks.protection.outlook.com/?url=https%3A%2F%2Fjournals.plos.org%2Fplosone%2Fs%2Feditorial-and-peer-review-process%23loc-peer-review-history&data=05%7C01%7CMoon.Sungrim%40mayo.edu%7C44fe22cb3ac1437853de08dac7270263%7Ca25fff9c3f634fb29a8ad9bdd0321f9a%7C0%7C0%7C638041267598666228%7CUnknown%7CTWFpbGZsb3d8eyJWIjoiMC4wLjAwMDAiLCJQIjoiV2luMzIiLCJBTiI6Ik1haWwiLCJXVCI6Mn0%3D%7C3000%7C%7C%7C&sdata=iXlZpOPY91mpC3DiXG4gnzSyKI7hGAymzzXxArm6Vgk%3D&reserved=0)). If published, this will include your full peer review and any attached files.


If you choose “no”, your identity will remain anonymous but your review may still be made public.


**Do you want your identity to be public for this peer review?** For information about this choice, including consent withdrawal, please see our [Privacy Policy](https://nam12.safelinks.protection.outlook.com/?url=https%3A%2F%2Fwww.plos.org%2Fprivacy-policy&data=05%7C01%7CMoon.Sungrim%40mayo.edu%7C44fe22cb3ac1437853de08dac7270263%7Ca25fff9c3f634fb29a8ad9bdd0321f9a%7C0%7C0%7C638041267598666228%7CUnknown%7CTWFpbGZsb3d8eyJWIjoiMC4wLjAwMDAiLCJQIjoiV2luMzIiLCJBTiI6Ik1haWwiLCJXVCI6Mn0%3D%7C3000%7C%7C%7C&sdata=GgsiXonVDgbCrsiZSVdwHKBFFelDZ1hR9xNffVgUwCA%3D&reserved=0).

Reviewer #1: No

Reviewer #2: No

[NOTE: If reviewer comments were submitted as an attachment file, they will be attached to this email and accessible via the submission site. Please log into your account, locate the manuscript record, and check for the action link "View Attachments". If this link does not appear, there are no attachment files.]

While revising your submission, please upload your figure files to the Preflight Analysis and Conversion Engine (PACE) digital diagnostic tool, [https://pacev2.apexcovantage.com/](https://nam12.safelinks.protection.outlook.com/?url=https%3A%2F%2Fpacev2.apexcovantage.com%2F&data=05%7C01%7CMoon.Sungrim%40mayo.edu%7C44fe22cb3ac1437853de08dac7270263%7Ca25fff9c3f634fb29a8ad9bdd0321f9a%7C0%7C0%7C638041267598666228%7CUnknown%7CTWFpbGZsb3d8eyJWIjoiMC4wLjAwMDAiLCJQIjoiV2luMzIiLCJBTiI6Ik1haWwiLCJXVCI6Mn0%3D%7C3000%7C%7C%7C&sdata=cskfSbUBc5WV8AJIzcpJYH5IccQK0QC0SVXSmAvL0n8%3D&reserved=0). PACE helps ensure that figures meet PLOS requirements. To use PACE, you must first register as a user. Registration is free. Then, login and navigate to the UPLOAD tab, where you will find detailed instructions on how to use the tool. If you encounter any issues or have any questions when using PACE, please email PLOS at [figures@plos.org](mailto:figures@plos.org). Please note that Supporting Information files do not need this step.
